# Triterpene derivative improves the renal function of streptozotocin-induced diabetic rats: a follow-up study on maslinic acid

**DOI:** 10.1080/0886022X.2019.1623818

**Published:** 2019-06-25

**Authors:** Blessing Nkazimulo Mkhwanazi, Fanie Retief van Heerden, Greanious Alfred Mavondo, Musa Vuyisile Mabandla, Cephas Tagumirwa Musabayane

**Affiliations:** a School of Agricultural, Earth & Environmental Sciences, University of KwaZulu-Natal, Scottsville, South Africa;; b School of Chemistry and Physics, University of KwaZulu-Natal, Scottsville, South Africa;; c Faculty of Medicine, National University of Science and Technology (NUST), Bulawayao, Zimbabwe;; d School of Laboratory Medicine and Medical Sciences, University of KwaZulu-Natal, Durban, South Africa

**Keywords:** Maslinic acid (MA), fractional excretion of Na^+^ (FENa), maslinic acid derivative, diabetes mellitus

## Abstract

**Introduction:** Reports indicate that oral administration of plant-derived maslinic acid (MA) exhibits hypoglycemic and renoprotective effects in streptozotocin (STZ)-induced diabetic rats. Challenges with triterpenes such as MA include low bioavailabilty which affects treatment efficacy in experimental animals. The goal of this study was to synthesize the MA derivative phenylhydrazine (PH-MA) in an effort to improve the efficacy of MA.

**Methods:** Separate groups of non-diabetic and STZ-induced diabetic rats (*n* = 6) were anesthetized and the jugular vein cannulated for the infusion of 0.077 M NaCl at 9 mL/h. The bladder was catheterized for collection the urine samples every 30 min. After 30.5 h equilibration period, consecutive 30 min urine collections were made over the subsequent 4 h of 1 h control, 1.5 h treatment, and 1.5 h recovery periods. PH-MA (22 µg/h) and MA (90 µg/h) were added during the treatment periods for analysis of proximal tubular Na^+^ handling, plasma aldosterone and arginine vasopressin in male Sprague-Dawley rats.

**Results:** Intravenous infusion of PH-MA (22 µg/h) and MA (90 µg/h) significantly (*p* ˂ .05) increased Na^+^ output, fractional excretion of Na^+^ (FENa) and lithium (FELi). Interestingly, like MA, PH-MA significantly (*p* ˂ .05) increased glomerular filtration rate (GFR) over the treatment period and decreased plasma aldosterone levels. Our findings indicate that PH-MA inhibited sodium reabsorption in the proximal and distal tubule as shown by increased FENa and low plasma aldosterone levels, respectively.

**Conclusions:** PH-MA is, therefore, a promising multitarget antidiabetic agent that may ameliorate kidney function of diabetic patients at a dose four times lower than the parent compound (MA).

## Introduction

Diabetes mellitus is a chronic disease that affects children and adolescents in both developing and developed countries [1,2]. Sustained hyperglycemia is a common sign of uncontrolled diabetes and over time may result in macro and microvascular complications [3,4]. Studies indicate that macrovascular complications such as diabetic nephropathy are the leading cause of chronic kidney disease in patients starting renal replacement therapy and is associated with increased cardiovascular mortality [5]. The onset of diabetic nephropathy is linked to increased expression of glucose transporters (GLUTs) and sodium/glucose cotransporter 2 (SGLT-2) in the kidney leading to reabsorption of glucose back to the systemic circulation [6]. Following this is a sequel of events, which include a decrease in glomerular filtration rate (GFR), sodium (Na^+^) retention and increased blood pressure in diabetic animals [7,8]. Retention of Na^+^ is attributed to increased expression of transporters such as sodium hydrogen transporter 3 (NHE3) and epithelium sodium channels (ENaC) that are abundant in the proximal and distal tubules of the nephron respectively [9,10]. Therefore, to prevent the onset of diabetic nephropathy focus should be on tight glycaemic control and prevention of Na^+^ reabsorption in the kidneys. To date, no treatment can provide these synergistic effects. Our studies reported that *Syzygium* spp-derived triterpenes, oleanolic acid (OA) [11,12], and maslinic (MA) [13] lower blood glucose concentration and ameliorate kidney function in streptozotocin (STZ)-induced diabetic rats. MA (2α,3β)-2,3-dihydroxyolean-12-en-28-oic acid, is one of the promising triterpenoids displaying a wide range of pharmacological properties including anticancer [14], anti-inflammatory [15], antidiabetic, and renoprotective effects [13]. The absence of adverse effects demonstrated by previous studies on maslinic acid (MA) constituted a promising starting point for its future use as a nutraceutical due to the biological activities described for this pentacyclic triterpene [16]. The mechanism for blood glucose lowering properties include glycogen phosphorylase [17] and protein tyrosine phosphatase (PTP1B) [18] inhibition. Of interest in our study is enhancing the efficacy of MA by increasing its potency as a PTP1B inhibitor [18,19]. Studies showed that incorporation of a heterocyclic ring in the carbon-2 and carbon-3 position enhanced the efficacy of MA 6-fold as a (PTP1B) inhibitor [18]. Consequently, we introduced a phenylhydrazine (PH) in C-2 and C-3 position of the parent compound to improve the efficacy of MA as a PTP1B inhibitor. Guided by this fundamental observation, we hypothesized that the MA derivative containing PH might possess more potency compared to lead MA. Accordingly, this study was designed to determine whether triterpene derivative (PH-MA) could improve the impaired renal fluid and electrolyte handling often seen in diabetic animals.

## Materials and methods

### Drugs and chemicals

All drugs used were sourced from standard pharmaceutical suppliers. All other chemicals, which were of analytical grade quality, were purchased from standard commercial suppliers.

### Synthesis of the phenylhydrazine derivative of maslinic acid (PH-MA)

#### Oxidation of OA

**Figure 1.**
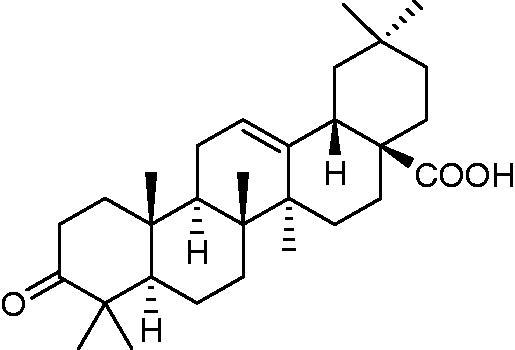
Chemical structure of oxidized oleanolic acid.

OA was used as the precursor material for the synthesis of the PH-MA triterpene derivative. OA was isolated from clove flower buds using our well-established protocol [11,12]. Oxidation of OA was performed as described by Zhang et al. [20]. A suspension of OA (1.0 g, 2.2 mmol) in 10 mL dichloromethane-acetone (1:1) was cooled to 5 °C and a solution of Jones reagent (1.2 mL, 5 equiv) was added dropwise over 30 min and the reaction was allowed to run for 1 h until the color turned dark brown. Isopropanol (10 mL) and H_2_O (15 mL) were added to the reaction mixture. The reaction mixture was then stirred at room temperature for 15 min. H_2_O and CH_2_Cl_2_ were added to the mixture and the layers were separated. The organic phase was washed with brine and the solvent removed on a rotavapor to give 0.90 g of oxidized OA (Figure 1). The pure product of oxidized OA (Figure 1) was obtained by silica gel chromatography (hexane-: ethyl acetate, 7:3) and was recrystallized from chloroform-methanol (1:1).

#### Phenylhydrazine

**Figure 2.**
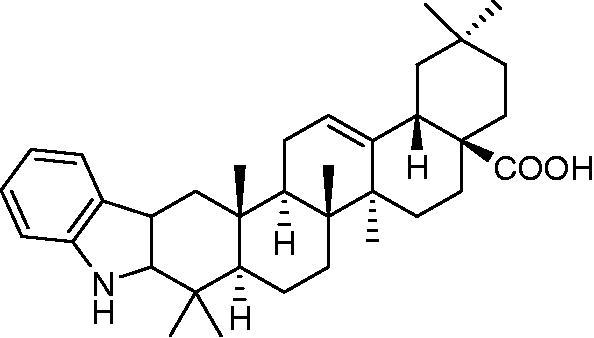
Chemical structure of the phenylhydrazine derivative (PH-MA).

Fischer indole synthesis was performed according to a method described in Alonso et al. [21]. Briefly, a mixture of the ketone of OA (1 g, 2.2 mmol), PH (0.8 mL 0.9 mmol), and glacial acetic acid (5 mL) was heated at reflux under nitrogen for 1 h. During this period, the color changed from colorless to bright yellow. The reaction mixture was pipetted into distilled water (50 mL) and extracted with ether (4 × 20 mL). The combined ether extracts were washed with 5% aqueous NaOH (2 × 20 mL) and brine (2 × 20 mL) followed by drying over Na_2_SO_4_. The combined extract was then concentrated *in vacuo* resulting in the formation of a solid yellow product. Chromatography over silica gel and elution with hexane-ethyl acetate (7:3) resulted in the isolation of the indole (Figure 2) (86%) as a yellow solid.

### Animal experiments

#### Animals

Male Sprague-Dawley rats weighing 250–300 g were obtained from the Biomedical Research Unit (BRU) of the University of KwaZulu-Natal (Westville campus). The animals were kept under maintained laboratory conditions of constant temperature (22 ± 1 °C); CO_2_ ( < 5000 ppm,) humidity of 55 ± 5% and illumination (12 h light/dark cycles). The animals had full access to food standard rat chow (Meadows Feeds, Pietermaritzburg, South Africa) and water. All experiments and protocols used in this study were reviewed and approved by the animal ethics committee of the University of KwaZulu-Natal (UKZN) with ethical clearance numbers 002/13/Animal and 029/14/Animal.

#### Induction of diabetes

Diabetes was induced with a single intraperitoneal injection of STZ (60 mg/kg) dissolved in 0.1 M citrate buffer pH 6.3 [13,22,23]. Control animals were injected with the vehicle (citrate buffer). Animals exhibiting glucosuria 24 h later following testing using urine strips (Rapidmed Diagnostics, Sandton, South Africa) were considered diabetic. The blood glucose concentration of 20 mmol/L measured one week later was considered to reflect a stable diabetic state.

#### Experimental design

Animal groups (*n* = 6 per group) were divided according to whether they were diabetic or non-diabetic.

The groups are as follows:

Non-diabetic control: infused with saline containing creatinine (0.15 µg/mL).
Diabetic control: infused with saline containing creatinine (0.15 µg/mL).
Non-diabetic treated: infused with 22 µg/h of PH-MA or MA containing creatinine (0.15 µg/mL).
Diabetic treated: infused with 22 µg/h of PH-MA or MA containing creatinine (0.15 µg/mL).

#### Proximal tubular function

Sodium (Na^+^) reabsorption in the proximal tubule and by implication in the distal nephron was assessed through measurement of lithium clearance (C_Li_) [24]. The effects of PH-MA treatment on renal function were investigated in separate groups of anesthetized non-diabetic and STZ-induced diabetic rats (*n* = 6 in each group). The rats were fed standard rodent chow supplemented with lithium chloride (12 mmol/kg dry weight) for 48 h prior to experimentation in order to raise plasma lithium to measurable concentrations without affecting renal sodium or water excretion [25]. Subsequently, renal clearance studies were conducted in inactin-anesthetized (0.11 g/kg) hypotonic saline infused non-diabetic and STZ-induced rats, a model that has been extensively used in our laboratory [7,13]. Briefly, the right jugular vein was cannulated to allow a continuous intravenous infusion of hypotonic saline (0.077 M NaCl) at 9 mL/h (Harvard syringe infusion Pump 22, Harvard Apparatus, Holliston, MA). A catheter was inserted into the left carotid artery for withdrawal of blood samples (Statham MLT 0380, Ad Instruments, Bella Vista NSW, Australia), compatible with PowerLab System ML410/W (Bella Vista NSW, Australia). The urinary bladder was also cannulated *via* an incision in the lower abdomen for the collection of urine samples. Animals were given a priming dose of creatinine (3 µg in 0.3 mL 0.077 M NaCl) before being placed on a continuous infusion of 0.077 M NaCl containing creatinine (0.15 µg/mL) running at 9 mL/h to allow calculation of creatinine clearance as a measure of GFR. After a 3.5 h equilibration period, blood samples (200 μL) were drawn at 1 h intervals and urine was collected every 30 min over 4 h for the study. The collection process was as follows, 1 h for control, 1.5 h treatment and 1.5 h recovery periods for measurement of electrolyte and clearance marker concentrations. The infusate containing MA (22 µg/h) was introduced during the treatment period.

#### Arginine vasopressin (AVP) assay

A standard enzymatic method was used to determine plasma AVP concentrations on non-diabetic and STZ-induced diabetic rats treated with PH-MA. The assays were performed on an Arg^8^-Vasopressin ELISA Kit, using reagents purchased from the manufacturer (Abcam, Cambridge, MA). The lower and upper limits of detection were 4–789 pmol L^−1^, respectively. The intra-assay analytical coefficient of variation ranged from 9.9 to 12.6% and the inter-assay coefficient of variation from 6.0 to 8.5%.

#### Aldosterone assay

Plasma aldosterone concentration was measured from blood samples collected from treated and untreated groups of non-diabetic and STZ-induced diabetic rats using an aldosterone ELISA kit (Cusabio Biotech Wuhan, Hubei, China). The lower and upper limits of detection were 3 pmol L^−1^ and 923 pmol L^−1^, respectively. The intra-assay analytical coefficient of variation ranged from 5.9 to 10.6% and the inter-assay coefficient variation from 6.0 to 8.5%.

#### Urinalysis

Urine flow was determined gravimetrically. Na^+^, K^+^, Cl^−^ and creatinine were analyzed using the Beckman Coulter Counter (Synchron CX3 Clinical Systems, Fullerton, CA) with commercial diagnostic kits from Beckman Coulter, Dublin Ireland. Lithium was determined using flame emission spectroscopy at 670.8 nm (Optima 2100 DV, Perkin Elmer, Shelton, CT) using a modified procedure that has been previously described in our laboratory [7,13]. Fractional excretions (FE) rates of Na^+^ (Fe_Na_) and Li (Fe_Li_) were determined simultaneously. C_Li_ was used as a marker for the output of Na^+^ from the proximal tubules [26]. Renal clearances (C) and fractional excretions (FE) were calculated using the standard formulae C = U × V/P and FE = C/GFR, where U is the urinary concentration, V is the urine flow rate and P is the plasma concentration. GFR, as assessed by creatinine clearance was calculated at 1 h intervals in anesthetized rats using the standard formulae for measurement of plasma and urinary concentrations of creatinine and urine flow rate.

### Statistical analysis

All data were expressed as means ± standard error of means (SEM). Statistical comparison of the differences between the control means and experimental groups was performed with GraphPad InStat Software version 5.00 (GraphPad Software, San Diego, CA), using one-way analysis of variance (ANOVA), followed by Tukey–Kramer multiple comparison test. A value of *p* ˂ .05 was considered significant.

## Results

^1^H NMR data below depicts different proton positioning for the phenylhydrazine (PH-MA) derivative dissolved in deuterated chloroform. ^1^H NMR CDCl_3_, δ_H_ 0.85. 0.90, 0.94, 1.16, 1.17, 1.24, 1.27 (each 3 H, *s*), 2.21 (IH, *d*, *J* = 14.5), 2.77 (1 H, *d*, *J* = 14.7), 2.87 (1 H, *m*), 5.28 (1 H, *s*), 7.05 (1 H, *m*), 7.10 (1 H, *m*), 7.29 (1 H, *d*, *J* = 7.8), 7.40 (1 H, *d*, *J* = 7.5), 7.69 (1 H, *s*, NH). The NMR spectroscopy is shown under the Supplementary data. A total yield of 80% was obtained for the PH derivative, and yellow crystals were obtained following recrystallization from methanol.

### Effects of PH-MA on electrolyte handling

**Figure 3.**
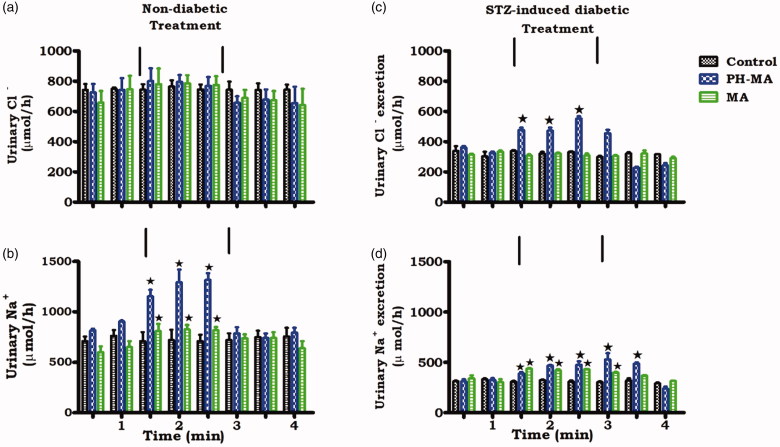
Acute effects of PH-MA administration on urinary Na^+^ and Cl^−^ excretion rate in non-diabetic rats (a–b) and STZ-induced diabetic rats (c–d). PH-MA was infused at 22 µg/h for 1.5 h during the treatment period. Values are presented as means for each 30 min collection; vertical bars indicate SEM of means (*n* = 6 rats in each group). ^★^*p* < .05 by comparison with control animals.

In Figure 3 the effects of PH-MA on urinary Na^+^ and Cl^−^ output in anesthetized rats is compared with the respective controls. The mean Na^+^ excretion rates of untreated STZ-induced diabetic rats ranged from 290 to 313 mmol h^−1^ over the 4 h experimental period, values which were significantly (*p* < .05) lower than those of control non-diabetic animals (range 711–756 mmol h^−1^). Infusion of PH-MA at 22 µg/h increased Na^+^ and Cl^−^ excretion in non-diabetic and STZ-induced diabetic rats (Figure 3). PH-MA, however, did not cause a significant change in urine flow rate and urinary K^+^ excretion (data not shown).

### Glomerular filtration rate (GFR)

**Figure 4.**
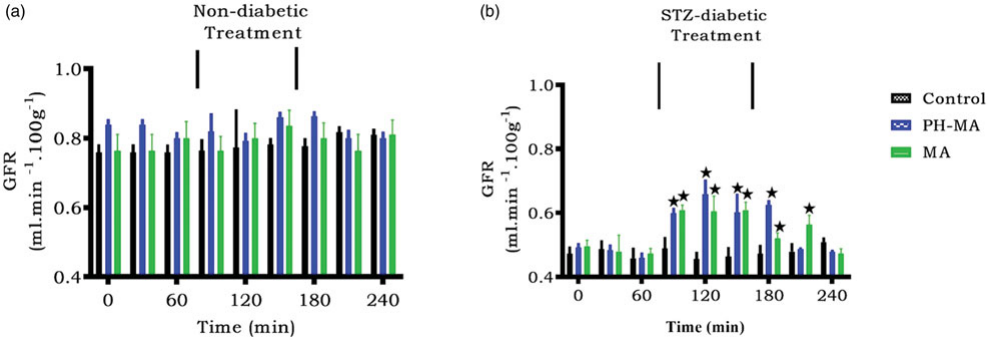
Acute effects of PH-MA administration on GFR rate in non-diabetic (a) and STZ-induced diabetic (b) rats. PH-MA was infused at 22 µg/h for 1.5 h during the treatment period. Values are presented as means for each 30 min collection; vertical bars indicate SEM of means (*n* = 6 rats in each group). ^★^*p* < .05 by comparison with control animals.

Figure 4 indicates that the GFR values of untreated diabetic rats were lower than the control non-diabetic rats. PH-MA administration for 1.5 h significantly increased GFR in STZ-induced diabetic rats (Figure 4), however, no change was observed in the non-diabetic animals.

### Renal clearance

**Figure 5.**
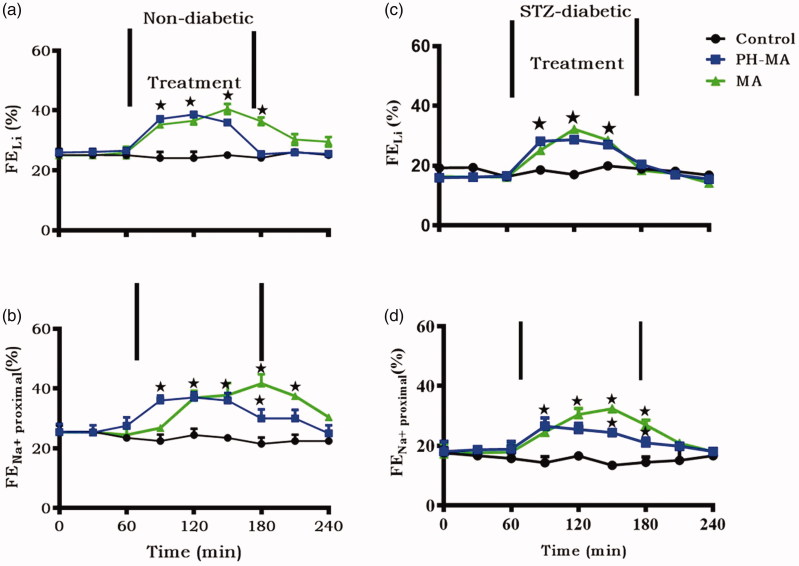
Effects of intravenously infused PH-MA on FE_Na+_ and FE_Li+_ in non-diabetic rats (a–b) and STZ-induced diabetic (c–d) (*n* = 6 in each group). Values are presented as means, and vertical bars indicate SEM (*n* = 6 in each group). ^★^*p* < .05 by comparison with respective control.

The effects of PH-MA on proximal tubular Na^+^ clearance were estimated by comparing renal lithium clearance (FE_Li_^+^) between anesthetized non-diabetic, STZ-induced diabetic controls and PH-MA-treated rats. Infusion of PH-MA (22 µg/h) for 1.5 h significantly (*p* < .05) increased fractional excretion rates of sodium (FE_Na+_) and lithium (FE_Li+_) in non-diabetic and STZ-induced diabetic rats when compared to the respective controls (Figure 5).

### AVP and aldosterone

Table 1 shows the concentration of plasma aldosterone and AVP measured in non-diabetic and STZ-diabetic rats following 1.5 h treatment period. Plasma AVP levels of the untreated STZ-diabetic rats were elevated when compared to non-diabetic controls, *p < .05 (Table 1). Aldosterone concentration was also elevated in the untreated STZ-induced diabetic rats when compared to non-diabetic controls, *p < .05 (Table 1). PH-MA treatment decreased aldosterone concentration in the STZ-induced diabetic rats when compared to the diabetic control group **p < .05 (Table 1).

**Table 1.** Plasma hormone concentration in rats after MA or PH-MA administration for 90 min of treatment.

Groups	AVP (pmol/L)	Aldosterone (nm/L)
Non-diabetic control	2.82 ± 0.47	0.78 ± 0.04
Non-diabetic MA treated	2.38 ± 0.21	0.70 ± 0.01
Non-diabetic PH-MA treated	2.44 ± 0.10	0.75 ± 0.07
STZ-diabetic control	4.45 ± 0.24*	1.45 ± 0.03*^**^^**^
STZ-diabetic MA treated	4.00 ± 0.26	0.79 ± 0.05^*^**
STZ-diabetic PH-MA treated	4.42 ± 0.33	0.80 ± 0.07**

* *p* < .05 STZ-diabetic controls compared to non-diabetic controls.

** *p* < 0.05 STZ-diabetic treated with PH-MA or MA compared to diabetic controls.

## Discussion

This study investigated the effects of a triterpene derivative on the renal function of non-diabetic and STZ-diabetic rats with the hope of providing evidence for the possible use of this compound in the management of renal complications in diabetes. Derivatives are generally synthesized based on the known triterpene active sites. Several triterpene derivatives have been synthesized in an effort to improve the water solubility of MA. However, the results were disappointing as the incorporation of hydrophilic groups on carbon 28 significantly decreased the potency of MA [17]. Our current research, therefore, focused on synthesizing an MA derivative with the aim to improve the efficacy of this triterpenes as a protein tyrosine phosphatase inhibitor. Fischer indole synthesis [18,27] resulted in a compound (PH-MA) with a ^1^H NMR spectrum comparable to the indole synthesized by Finlay et al. [28].

Experimental diabetes was induced with a single intraperitoneal STZ injection which selectively destroys secretion of insulin from pancreatic β-cells [29,30]. STZ-induced diabetic rats exhibited low urinary, GRF, Na^+^, and Cl^−^ excretion rates in comparison to non-diabetic rats. Indeed, previous studies have validated that STZ-induced diabetic animals tend to retain Na^+^_,_ whilst elevating plasma aldosterone concentration [7,31]. Our finding, however, indicates that PH-MA like MA reverses the reabsorption of Na^+^ and Cl^−^ suggesting its beneficial effects in ameliorating kidney function in diabetic animals. Triterpenes such as OA and MA have previously been shown to inhibit Na^+^ reabsorption in the proximal and distal tubules at 90 µg/h infusion [11,13]. We used the lower dose (22 µg/h) to minimize any potential toxicity of this triterpene derivative, as infusion of this derivative on an animal model was done for the first time. The interesting observation is that PH-MA exerted pronounced effects at 22 µg/h, a dose that was four times lower than the parent compound. In an attempt to elucidate the mechanism of action, we evaluated fractional excretion of Na^+^ (FENa) and hormones associated with natriuresis. Our results indeed showed increased fractional excretion of sodium and lithium suggesting that Na^+^ handling was in part due to proximal tubular reabsorption. The exact mechanism(s) and transporters that are involved in impaired proximal tubular Na^+^ reabsorption could not be elucidated in this study. However, the proximal tubules reabsorb about 70% of total filtered Na^+^ [32]. The reabsorption involves the coupling of Na^+^ entry *via* the sodium-proton exchanger type 3 (NHE3) transporters whereby the Na^+^ extrusion primarily occurs through Na^+^-K^+^-ATPase [33]. Another important transporter found in the proximal tubule is the sodium-bicarbonate cotransporter (NBCe1) which mediates Na^+^ exit from the tubular cells [34]. Therefore, increased FENa by PH-MA may be attributed to inhibiting the expression of apical NHE3 and basolateral Na^+^-K^+^-ATPase stimulated basolateral NBCe1 transporter which accounts for the reabsorption of Na^+^ from the proximal tubular [35]. Previous studies by Riquier et al. in 2009 support our findings as they showed a correlation between Na^+^ reabsorption and NHE3 expression [36]. Moreover, drugs like a-adrenoceptor agonists and inhibitory parathyroid hormone (PTH) which regulate both NHE3 and Na^+^ reabsorption showed the involvement of this transporter in Na^+^ handling suggesting our compound could in part be involved in inhibiting this transporter [37].

To investigate the involvement of other factors contributing to electrolyte handling, we evaluated the influence of PH-MA on plasma aldosterone. Aldosterone regulates extracellular fluid volume by increasing sodium reabsorption in the distal tubule and the cortical collecting duct of the kidney [38,39]. Recent studies have demonstrated that aldosterone also regulates sodium transport in the proximal tubule by increasing expression of NH_3_ expression in the kidney [40]. Our PH-MA results were similar to that of amlodipine, which decreased plasma aldosterone, attenuated oxidative stress thus ameliorating diabetic induced renal injury [41]. Matavelli et al. demonstrated a relationship between the inhibition of aldosterone, reduced oxidative stress and improved kidney function on diabetic rats [41]. Our findings may, therefore, suggest that PH-MA and MA reduce aldosterone through amelioration of oxidative stress, hence the administration of triterpenes did not alter plasma aldosterone on non-diabetic rats. The above speculative results warrant further investigation to elucidate the association between the aldosterone and the oxidative stress. Results presented in this article are in agreement with a previous study which has shown that heterocyclic indole derivatives improve the effects of natural products or parent compounds [42].

## Conclusions

Our data render support to the hypothesis that PH-MA could improve impaired electrolyte handling seen in diabetic animals. Intravenous infusion of PH-MA (22 µg/h) improved fractional excretion of sodium and lithium, indicating that PH-MA inhibited sodium reabsorption in the proximal tubule. A decrease in plasma aldosterone indicates that PH-MA does not only influence sodium handling in the proximal tubule but also through the distal tubule and collecting duct as well. PH-MA everted the low GFR seen in diabetic animals due to thickened glomerular basement membrane, indicating the ability of PH-MA to ameliorate kidney function. These results suggest that PH-MA may be a promising anti-diabetic agent administered at lower doses than the parent compound MA. The findings in this study have opened a wide gap of areas that warrant further investigation, these include solubility, pharmacokinetics, and long-term (5-weeks) studies of PH-MA compared to the parent compound.

## Supplementary Material

Supplementary Material
