# Supplementary material for: Triterpene derivative improves the renal function of streptozotocin-induced diabetic rats: a follow-up study on maslinic acid
Source: Ren Fail. 2019 Jun 25;41(1):547–54. doi: 10.1080/0886022X.2019.1623818 (PMC6598493; doi:10.1080/0886022X.2019.1623818)
Supplement: Supplementary Material [file IRNF_A_1623818_SM2601.docx]

**^1^HNMR of phenyl hydrazine-MA derivative**

^1^H NMR data below depicts different proton positioning for the phenylhydrazine (PH-MA) derivative dissolved in deuterated chloroform. ^1^H NMR CDCl_3_, δ_H_ 0.85. 0.90, 0.94, 1.16, 1.17, 1.24, 1.27 (each 3H, *s*), 2.21 (IH, *d*, *J* = 14.5), 2.77 (1H, *d*, *J* = 14.7), 2.87 (1H, *m*), 5.28 (1H, *s*), 7.05 (1H, *m*), 7.10 (1H,*m*), 7.29 (1H, *d*, *J* = 7.8), 7.40 (1H, *d*,  *J* = 7.5), 7.69 (1H, *s*, NH). A total yield of 80% was obtained for the phenylhydrazine derivative, and yellow crystals were obtained following recrystallisation from methanol

**
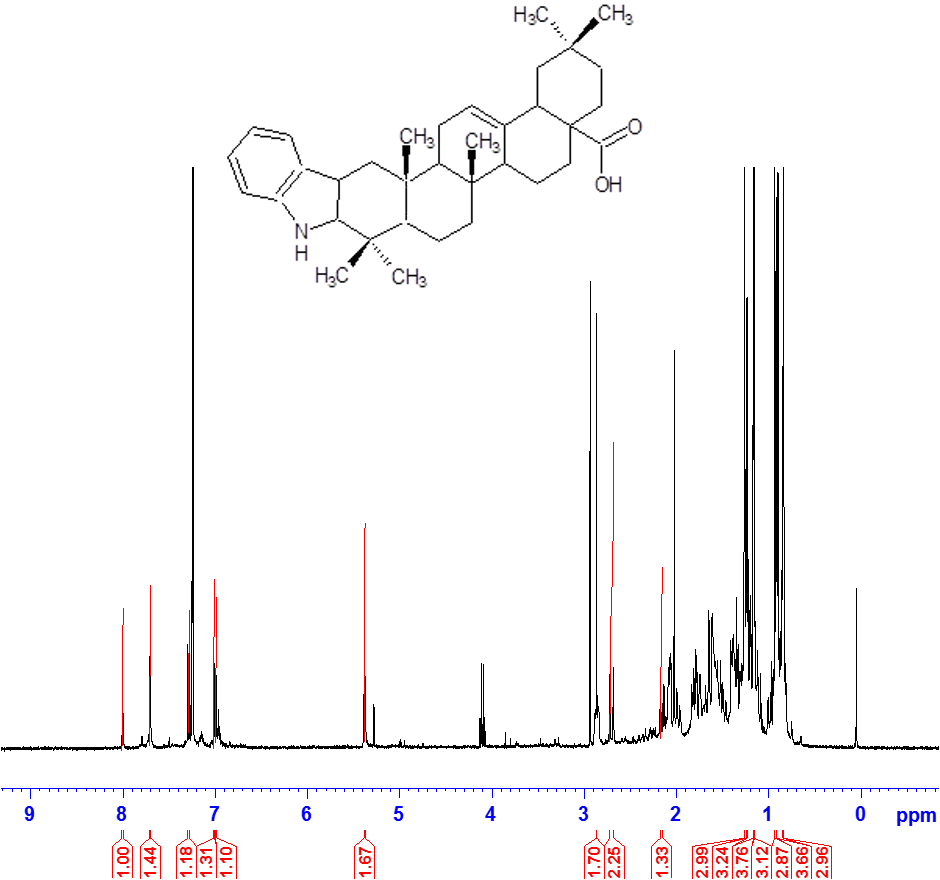
**
